# Neutrophil/Lymphocyte Ratio (NLR) as a Predictive Marker for p16 Positivity and Cervical Cancer Progression: Insights from the SCOPE Study

**DOI:** 10.3390/cancers17060921

**Published:** 2025-03-08

**Authors:** Zsófia Tóth, Lotti Lőczi, Barbara Sebők, Petra Merkely, Emese Keszthelyi, Balázs Lintner, Nándor Ács, Attila Keszthelyi, Szabolcs Várbíró, Richárd Tóth, Márton Keszthelyi

**Affiliations:** 1 Department of Obstetrics and Gynecology, Semmelweis University, 1082 Budapest, Hungary; toth.zsofia99@stud.semmelweis.hu (Z.T.); keszthelyi.lotti.lucia@semmelweis.hu (L.L.); merkely.petra@semmelweis.hu (P.M.); keszthelyibp@gmail.com (E.K.); lintner.balazs.zoltan@semmelweis.hu (B.L.); acs.nandor@semmelweis.hu (N.Á.); varbiroszabolcs@gmail.com (S.V.); toth.richard@semmelweis.hu (R.T.); 2 Workgroup of Research Management, Doctoral School, Semmelweis University, 1085 Budapest, Hungary; sebok.barbara23@gmail.com; 3 Department of Urology, Semmelweis University, 1082 Budapest, Hungary; keszthelyi.attila@semmelweis.hu; 4 Department of Obstetrics and Gynecology, University of Szeged, 6725 Szeged, Hungary

**Keywords:** cervical cancer, human papillomavirus (HPV), neutrophil/lymphocyte ratio (NLR), p16 positivity, biomarkers, systemic inflammation

## Abstract

**Background:** Cervical cancer, primarily driven by persistent high-risk human papillomavirus (HPV) infections, remains a significant global health challenge. Systemic inflammatory markers, such as neutrophil/lymphocyte ratio (NLR), platelet/lymphocyte ratio (PLR), and lymphocyte/monocyte ratio (LMR), may reflect disease progression. This study examines the association between these markers and p16 positivity in cervical intraepithelial neoplasia (CIN) cases. **Methods:** This retrospective analysis included 395 patients undergoing LEEP conization. Data on HPV status, p16 immunostaining, and hematological parameters were collected. Statistical analyses, including Mann–Whitney U and chi-square tests, assessed relationships between markers and outcomes, with significance set at *p* < 0.05. **Results:** Elevated NLR was significantly associated with p16 positivity (*p* = 0.011) and HPV DNA positivity (*p* = 0.04). HPV-positive individuals showed higher mean NLR (2.15) compared to HPV-negative individuals (1.61). Receiver operating characteristic (ROC) analysis demonstrated moderate diagnostic accuracy for NLR (AUC = 0.610), highlighting its potential as a biomarker. No significant associations were observed for PLR or LMR with p16 positivity. These findings suggest systemic inflammation, indicated by NLR, contributes to HPV persistence and CIN progression. **Conclusions:** NLR is a valuable prognostic biomarker for HPV-related cervical disease, correlating with both p16 and HPV DNA positivity. Incorporating hematological and immunohistochemical markers may enhance personalized cervical cancer management.

## Simple Summary

Cervical cancer is a major global health concern, primarily caused by persistent high-risk human papillomavirus (HPV) infections. Identifying reliable biomarkers for early disease detection and progression monitoring is crucial. This study examines the relationship between systemic inflammatory markers—neutrophil/lymphocyte ratio (NLR), platelet/lymphocyte ratio (PLR), and lymphocyte/monocyte ratio (LMR)—and p16 positivity, a key biomarker for HPV-related cervical disease. Our analysis of 395 patients undergoing LEEP conization found that elevated NLR levels were significantly associated with both p16 and HPV DNA positivity, suggesting a link between systemic inflammation and disease progression. These findings highlight the potential of NLR as an accessible and cost-effective prognostic marker, offering valuable insights for improving cervical cancer screening and management. By integrating hematological and immunohistochemical markers, clinicians may enhance risk stratification and personalized patient care.

## 1. Introduction

Cervical cancer is the fourth most common oncological disease among women worldwide, with its highest incidence typically occurring between the ages of 30 and 35 [1]. Despite advancements in screening methods, the disease is often diagnosed at advanced stages due to inadequate early detection and inconsistent follow-up care. Persistent infection with high-risk human papillomavirus (HPV) is the primary etiological factor, contributing to over 95% of cervical cancer cases [2]. This sexually transmitted virus infects the epithelium of the uterine cervix, vagina, and vulva in women; the penis in men; and skin and oropharyngeal epithelia in both sexes, progressively leading to malignant transformations in susceptible tissues.

Cervical intraepithelial neoplasia (CIN) is a precancerous condition characterized by abnormal epithelial cell growth in the epithelium of the uterine cervix. CIN is classified into three grades of severity—CIN I, CIN II, and CIN III—depending on the thickness affected by dysplastic cells in the epithelial layer. Loop electrosurgical excision procedure (LEEP) conization is a widely utilized method for managing CIN, offering both diagnostic and therapeutic benefits by excising abnormal tissue. While effective, LEEP conization may reveal lesions more advanced than initially predicted by cytology, underscoring the limitations of traditional cytological screening.

In the context of cervical cancer, p16 positivity serves as a crucial biomarker linked to HPV-related malignancies in cervical samples. Overexpression of p16, a tumor suppressor protein, is induced by high-risk HPV types, signifying active oncogenic transformation [3,4]. The expression of p16INK4a (p16) has been recognized as a significant biomarker in assessing cervical intraepithelial neoplasia (CIN) and predicting the progression of cervical lesions. p16 positivity is often associated with high-risk human papillomavirus (HR-HPV) infections, particularly the E7 oncoprotein, which disrupts the retinoblastoma protein pathway, leading to increased levels of p16 expression in affected tissues [5]. The use of p16 immunohistochemistry (IHC) has been integrated into clinical practice to enhance the prognostic value of cytologic screening, particularly in distinguishing between low-grade and high-grade lesions [6].

Adjunctive tests, such as p16/Ki-67 dual staining, have been integrated into clinical practice to enhance diagnostic accuracy [7]. p16/Ki-67 dual staining detects the simultaneous presence of two biomarkers, p16 and Ki-67, within individual cells, which serve as strong indicators of cellular transformation associated with cervical cancer risk. By utilizing the same sample as HPV or Pap testing, p16/Ki-67 dual staining facilitates timely and efficient patient management [8]. These tests help to identify high-grade lesions earlier, considering the lesion’s high potential for progression. However, even with these advancements, diagnostic gaps persist, necessitating additional biomarkers to assess disease severity and to predict outcomes.

The neutrophil/lymphocyte ratio (NLR) has emerged as a potential prognostic biomarker in oncology. NLR, calculated as the ratio of neutrophils to lymphocytes in peripheral blood, reflects the balance between acute inflammation and adaptive immune response [3]. Elevated NLR has been associated with the progression and prognosis of various cancers, including gastrointestinal, genito-urinary tract, lung, and breast cancer [9,10]. In hepatocellular carcinoma, a higher NLR correlates with poor prognosis, indicating systemic inflammation and impaired immune function as contributors to disease progression [11]. The normal range for NLR in a healthy adult population is established between 0.78 and 3.53 [12]. In addition to NLR, other hematological biomarkers such as the platelet/lymphocyte ratio (PLR) and the monocyte/lymphocyte ratio (MLR) have also been explored in oncology [13]. PLR has shown associations with inflammation and cancer progression, while MLR reflects monocyte-driven inflammatory processes [14,15]. However, NLR remains the most consistent and clinically relevant predictor of disease severity in cervical cancer [16].

This study explores the relationship between NLR, PLR, MLR, and p16 positivity in patients undergoing LEEP conization for cervical intraepithelial neoplasia. Specifically, we aim to determine whether hematologic markers correlate with p16 expression levels, thereby reflecting disease advancement. By examining this potential association, we seek to evaluate its utility as a prognostic biomarker and its implications for personalized management strategies in cervical cancer care.

## 2. Materials and Methods

### 2.1. Patients

**Figure 1:**
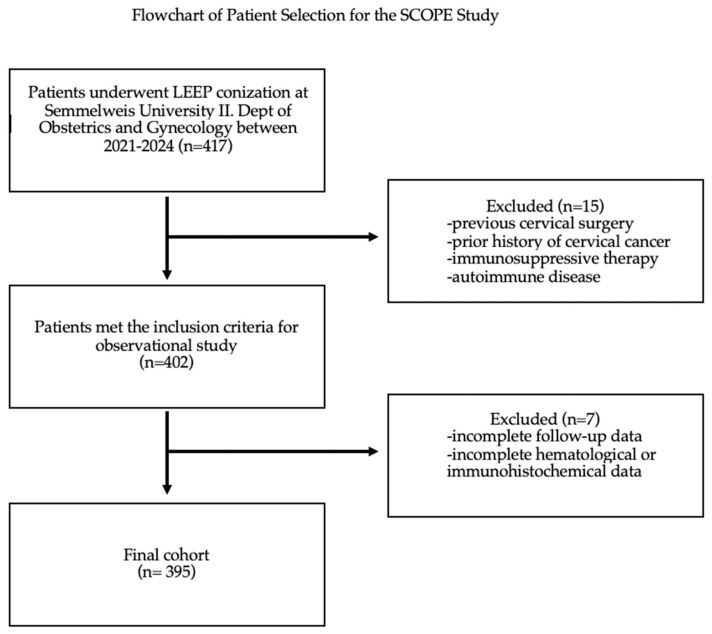
Flowchart of patient selection for the SCOPE Study. This flowchart illustrates the selection process for the patient cohort in the retrospective observational study.

This retrospective observational analysis of 395 patients, conducted as part of the SCOPE Study (Semmelweis University Conization and Inflammation Outcomes with Predictive Evaluation), involved a thorough chart review between 2021 and 2024 (Figure 1). The data collected covered a wide range of sociodemographic, gynecological, clinical, and laboratory variables to provide a holistic understanding of patient outcomes post-conization.

Patients were included in the study if they underwent LEEP conization at the Department of Obstetrics and Gynecology, Semmelweis University; had complete histopathological and hematological data, including NLR, PLR, MLR, and p16 immunohistochemical staining results; and were at least 18 years old at the time of the procedure.

Exclusion criteria were applied to patients who had a prior history of cervical cancer, previous cervical surgery that could have influenced histopathological or hematological findings, or were undergoing or had previously received immunosuppressive therapy. Patients diagnosed with autoimmune diseases that could affect inflammatory markers were also excluded, as were those with incomplete follow-up data or missing relevant clinical information. Additionally, any cases with incomplete hematological or immunohistochemical data necessary for statistical analysis were excluded.

### 2.2. Characteristics

The sociodemographic characteristics included the patients’ age, calculated by subtracting the year of birth from the year of the surgery. Additional factors included body weight, height, BMI, smoking status, alcohol consumption, known diabetes or hypertension, and the presence of immunological disorders affecting immune function.

All laboratory analyses were conducted in laboratories accredited by the National Accreditation Authority of Hungary, ensuring compliance with quality and accreditation standards. The laboratory parameters included inflammation-related biomarkers, such as neutrophil count, lymphocyte count, monocyte count, and platelet count. From these, specific ratios—neutrophil/lymphocyte ratio (NLR), monocyte/lymphocyte ratio (MLR), and platelet/lymphocyte ratio (PLR)—were calculated. All laboratory tests were performed within one month before surgery.

The screening of cervical dysplasia included the results of cervical cancer screening and HPV status, with a specific focus on the presence of high-risk HPV. Surgical data covered the outcomes of conization and histopathological findings from the conization procedure. These findings encompassed p16 positivity and the presence of glandular involvement. p16 immunohistochemical staining was performed on formalin-fixed, paraffin-embedded histological sections obtained from the LEEP conization specimens.

This study was ethically approved by the Institutional Review Board of Semmelweis University, (SE RKEB: 195/2024).

### 2.3. Data Management

Data for this retrospective analysis were collected and stored in a dedicated database designed for the SCOPE Study. The database included comprehensive patient records, encompassing sociodemographic, clinical, and laboratory variables. Before analysis, data integrity was ensured through rigorous checks for outliers and inconsistencies, utilizing boxplot visualizations to identify any extreme values that could potentially skew results. Missing data were systematically addressed according to predefined criteria, ensuring that the dataset remained robust for statistical evaluation.

### 2.4. Statistical Analysis

Statistical analyses were performed using IBM SPSS Statistics for Windows, Version 25.0 (Released 2017. IBM Corp., Armonk, NY, USA). Descriptive statistics, including mean, standard deviation, median, minimum, and maximum values, were calculated for all continuous variables to summarize the characteristics of the study population. The Mann–Whitney U test was employed to compare laboratory values and other variables between groups, particularly assessing differences in neutrophil/lymphocyte ratio (NLR), platelet/lymphocyte ratio (PLR), and lymphocyte/monocyte ratio (LMR) in relation to p16 positivity. A chi-square test was also utilized to evaluate categorical variables. Significance levels were set at *p* < 0.05.

To further explore the predictive capability of laboratory markers for p16 positivity, receiver operating characteristic (ROC) curves were constructed. This analysis provided insight into the sensitivity and specificity of NLR, PLR, and LMR as potential biomarkers in cervical cancer progression. The results were interpreted to determine optimal cutoff values for clinical application.

## 3. Results

### 3.1. Patient Characteristics

Participant characteristics are presented in Table 1. The median age of the 395 patients was 40 years (interquartile range: 23–78). The median BMI was 22.85 (interquartile range: 14.6–46.4). The median of NLR was 1.85 (interquartile range: 0.47–16.86), PLR was 8.89 (interquartile range: 3.3–184.2), and LMR was 4.34 (interquartile range: 0.32–18.5). Overall, 94.3% of patients were reported to have HPV positivity, and p16 positivity after conization was reported in 198 patients (79.8%). More than 90% of all patients reported no alcohol consumption, no known diabetes or hypertension, or any immunological disorders. Smoking was reported in 326 (83%) patients.

### 3.2. Relationship Between Laboratory Parameters and p16 Positivity

The relationship between p16 positivity identified in the conization sample and three hematological parameters—neutrophil/lymphocyte ratio (NLR), platelet/lymphocyte ratio (PLR), and lymphocyte/monocyte ratio (LMR)—was examined using the Mann–Whitney U test (Table 2). A significant association was found between p16 positivity and NLR (U = 3687.5, *p* = 0.011). No significant relationship was observed between p16 positivity and PLR (*p* = 0.366) and LMR (*p* = 0.145).

### 3.3. Laboratory Markers and Correlation of HPV DNA Status

The analysis of hematological parameters revealed differences between HPV DNA-positive and HPV DNA-negative groups (Table 3 and Table 4). The median neutrophil/lymphocyte ratio (NLR) was higher in the HPV DNA-positive group (median = 1.79) compared to the HPV DNA-negative group (median = 1.54). Similarly, the median platelet/lymphocyte ratio (PLR) was elevated in the HPV DNA-positive group (median = 9.08) relative to the HPV DNA-negative group (median = 7.64). In contrast, the median lymphocyte/monocyte ratio (LMR) was lower in the HPV DNA-positive group (median = 4.39) compared to the HPV DNA-negative group (median = 5.38). There was a significant association between HPV DNA positivity and NLR values only exceeding 1.31 (*p* = 0.04). These results suggest that higher NLR values are strongly associated with HPV DNA positivity, indicating a potential link between systemic inflammation and HPV infection status.

### 3.4. Diagnostic Performance

**Figure 2:**
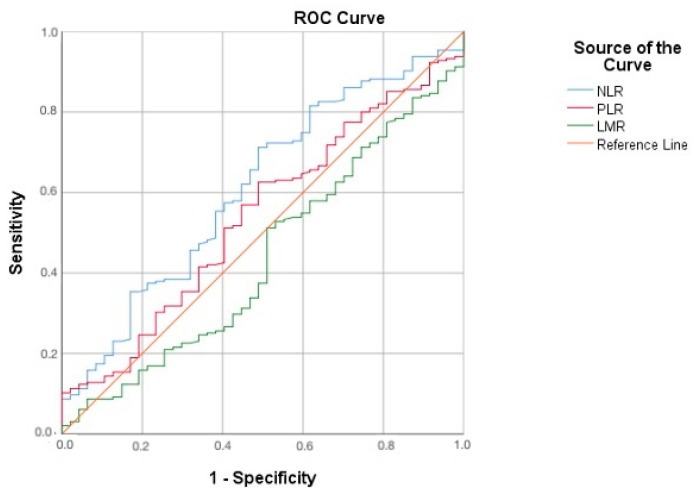
The ROC curve compares the diagnostic performance of the neutrophil/lymphocyte ratio (NLR), platelet/lymphocyte ratio (PLR), and lymphocyte/monocyte ratio (LMR) for identifying p16 positivity. Sensitivity (true positive rate) is plotted on the *y*-axis, while 1-specificity (false positive rate) is on the *x*-axis.

Receiver operating characteristic (ROC) analysis was performed to evaluate the diagnostic utility of NLR, PLR, and LMR in predicting p16 positivity. The area under the curve (AUC) for NLR was 0.610, demonstrating moderate diagnostic accuracy (Figure 2).

**Figure 3:**
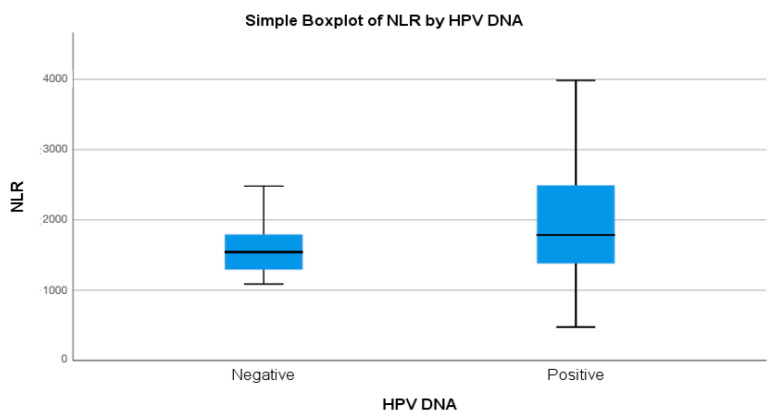
Boxplot showing the distribution of neutrophil/lymphocyte ratio (NLR) values by HPV DNA status. HPV DNA-positive individuals exhibit higher median NLR values compared to HPV DNA-negative individuals. This illustrates the significant association between elevated NLR values and HPV DNA positivity.

For a calculated optimal NLR cut-off value of ≥1.530, the maximal specificity was 51%, and the maximal sensitivity was 71% (Figure 3).

For PLR, the AUC was lower, at 0.540, reflecting weak discriminatory power. Similarly, LMR displayed limited diagnostic utility, with an AUC of 0.568. These values indicate that neither PLR nor LMR are reliable standalone markers for predicting p16 positivity compared to NLR.

### 3.5. Influencing Factors of the Findings

No significant correlations were found between laboratory markers (NLR, PLR, and LMR) and smoking status, alcohol consumption, diabetes, or immunological disorders among the patients. However, specific associations were observed with age and hypertension (Table 5).

Given the significant association between hypertension and NLR, a logistic regression analysis was conducted. The results of the logistic regression showed that NLR remained a statistically significant predictor of p16 positivity (B = 0.468, *p* = 0.026, Exp(B) = 1.596, 95% CI: 1.059–2.406), indicating that for each unit increase in NLR, the likelihood of p16 positivity increased by approximately 59.6%. In contrast, hypertension was not found to be a significant predictor (B = 0.541, *p* = 0.402, Exp(B) = 1.717, 95% CI: 0.485–6.076), suggesting that it does not independently influence the relationship between NLR and p16 positivity. The model’s overall predictive ability was low (Nagelkerke R^2^ = 0.055), indicating that NLR, while significant, explains only a small portion of the variability in p16 positivity. However, the model’s improvement with the inclusion of variables was statistically significant (χ^2^(2) = 8.671, *p* = 0.013).

These findings suggest that although hypertension is linked to higher NLR levels, it does not confound the relationship between NLR and p16 positivity. Thus, NLR remains an independent and significant predictor of p16 positivity in this population.

### 3.6. Validation Tests of HPV Positivity and p16 Positivity

Additional statistical tests were performed to ensure our histological findings’ validity. The chi-square analysis demonstrated a strong association between p16 positivity and HPV DNA status (x^2^ = 13.768, *p* = 0.000), supporting the accuracy of our histological data and the robustness of the statistical methods applied.

## 4. Discussion

The SCOPE study provides a comprehensive analysis of the relationship between systemic inflammatory markers, specifically the neutrophil/lymphocyte ratio (NLR), and p16 positivity in cervical intraepithelial neoplasia. By elucidating the interplay between systemic inflammation, human papillomavirus (HPV) infection, and the progression of cervical lesions, this research offers significant insights into the underlying pathophysiology and potential prognostic biomarkers in HPV-related cervical disease.

HPV infection remains a major challenge, as cervical cancer continues to be one of the most prevalent malignancies globally, particularly in developing countries [17]. While HPV vaccination provides robust protection, the prevalence of HPV genotypes shifts with age, with some types decreasing and others increasing in older women. Research such as Serrano et al.’s highlights the importance of understanding HPV persistence and its implications for risk stratification and management [18]. P16 immunostaining serves as a surrogate marker for transcriptionally active high-risk HPV infections, reflecting active oncogenic processes. Its sensitivity in predicting progression to CIN3 has been noted to reach 100%, with a similarly high negative predictive value, emphasizing its critical role in clinical decision-making [19,20].

In 1963, Virchow first established the connection between cancer and chronic inflammation [21]. Systemic inflammatory markers such as NLR, PLR, and LMR have become focal points in oncological research due to their diagnostic and prognostic potential [22,23]. Subsequent research has underscored the diagnostic efficiency of NLR, PLR, and LMR, particularly in endometrial carcinoma [24,25]. Among these, NLR stands out as the most reliable marker for monitoring oncological progressions [26]. The strong correlation between NLR and HPV DNA positivity and p16 positivity observed in our study reinforces the hypothesis that systemic inflammation, indicated by elevated NLR, facilitates the persistence of high-risk HPV and subsequent neoplastic transformation.

In this study, NLR was significantly associated with HPV DNA positivity (mean NLR for HPV-positive patients: 2.15; HPV-negative patients: 1.61, *p* = 0.04) and with p16 positivity (*p* = 0.011). These findings align with previous research, such as studies by Jun-Qiang Du et al., which highlighted the prognostic value of elevated NLR in early-stage cervical cancer. Neither PLR nor LMR showed significant associations with p16 positivity in our study (*p* = 0.366 and *p* = 0.145, respectively). Similarly, Lina Xu et al. observed significant differences in NLR values between CIN1, CIN2, and CIN3 groups, with higher NLR values associated with HPV16 persistence [27,28]. Despite the lack of statistical significance in our findings, including PLR and LMR helped confirm the specificity of NLR as the most robust marker of systemic inflammation in HPV-related cervical disease. This comprehensive analysis strengthens the argument that NLR has a more consistent association with both HPV DNA positivity and p16 positivity compared to other inflammatory markers.

Peripheral blood NLR measurements offer convenience and reproducibility, making them an auxiliary indicator for tumor prognosis.

These findings support earlier observations, such as those by Dominoni et al. who demonstrated that CIN2+ recurrences were significantly higher in patients with low NLR (<1.34) and that recurrence-free survival was greater in those with NLR ≥ 1.34 [29]. Similarly, studies by Zhai et al. and Origoni et al. have shown that elevated NLR values correlate with an increased risk of recurrence or residual disease, particularly in patients with high-grade squamous intraepithelial lesions [30,31]. These findings collectively suggest a stepwise increase in NLR values with advancing CIN grade and underscore the importance of NLR as a prognostic marker for transcriptionally active high-risk HPV infections. The ROC analysis of our study suggests that NLR outperforms PLR and LMR in its ability to differentiate p16-positive from p16-negative cases. However, even with moderate performance, NLR holds promise as a supportive diagnostic tool.

To ensure that these associations are independent of other conditions potentially elevating systemic inflammatory markers, we also examined the relationship between NLR, PLR, and LMR and factors such as smoking, alcohol consumption, diabetes, and immunological disorders. Our analysis revealed no significant correlations, indicating that the observed associations between systemic inflammation and CIN progression are likely independent of these confounding factors. This strengthens the robustness of our findings by demonstrating that the elevated inflammatory markers are primarily driven by HPV-related disease processes rather than other underlying inflammatory conditions.

The SCOPE study also highlights the challenges associated with defining universal cut-off values for NLR. For instance, while this study observed that all patients with NLR values above 1.31 were HPV-positive, no definitive threshold for normal NLR values could be established, reflecting variations reported in the literature. Future research should focus on standardizing these thresholds to enhance clinical applicability.

Our findings reinforce the link between systemic inflammation and active HPV infections, suggesting that higher NLR values reflect an inflammatory state conducive to viral persistence and progression to CIN or cervical cancer, and demonstrate a clear correlation between NLR and p16 positivity, a surrogate marker for transcriptionally active high-risk HPV. This connection emphasizes the role of systemic inflammation in driving HPV persistence and progression to malignant transformation.

### 4.1. Strengths and Limitations

This study offers a comprehensive evaluation of systemic inflammatory markers, particularly the neutrophil/lymphocyte ratio, in the context of HPV-related cervical intraepithelial neoplasia. By incorporating a large sample size of 395 patients, robust statistical analyses, and the integration of both hematological and immunohistochemical markers, the findings provide valuable insights into the prognostic potential of NLR. Furthermore, the use of receiver operating characteristic analysis strengthens the diagnostic utility of NLR by quantifying its sensitivity and specificity in predicting p16 positivity. The inclusion of real-world clinical data enhances the study’s applicability to everyday practice, emphasizing the utility of routine laboratory tests in cervical cancer management. As a retrospective study, the analysis is subject to inherent limitations, including potential selection and information biases. Unlike randomized controlled trials, this study lacks randomization and prospective data collection, which may impact the generalizability of the findings. Additionally, while the study identifies significant associations, causality cannot be established due to the study design.

### 4.2. Implication for Practice

NLR, as a cost-effective and widely accessible marker, provides an additional layer of diagnostic precision when combined with p16 immunostaining. This can enhance the early detection of high-grade lesions. Importantly, routine blood tests, including NLR, are universally available, require no additional cost, and are already part of standard preoperative assessments. In resource-limited settings, where advanced adjunctive tests such as p16/Ki-67 dual staining might not be affordable and diagnostic pathways may still heavily rely on the sensitivity of the Pap test alone, NLR emerges as a valuable, simple, repeatable, and promising tool. Its integration into cervical cancer screening and management protocols could aid in early disease detection, risk stratification, and treatment monitoring with minimal financial burden on healthcare systems. This highlights the clinical relevance of our findings, particularly in settings where cost-effective and accessible diagnostic solutions are most needed.

### 4.3. Future Perspective

Future perspectives include the need for longitudinal studies to evaluate the predictive accuracy of NLR over time and its role in guiding therapeutic decisions. Investigating interventions targeting systemic inflammation could also provide valuable insights into altering the disease trajectory of HPV-related cervical pathology.

## 5. Conclusions

This study highlights the critical interplay between systemic inflammation and molecular markers in cervical oncogenesis. The significant association between NLR and p16 positivity emphasizes the value of integrating systemic and local biomarkers to improve risk stratification and clinical management of HPV-related cervical lesions. By providing a cost-effective, non-invasive diagnostic adjunct, these findings pave the way for innovative approaches to cervical cancer prevention and care.

## Figures and Tables

**Table 1:** Characteristics of the sample. Categorical parameters are presented as *n*. Continuous data are presented as median (interquartile range).

Characteristics (*n* = 395)	N (Range or %)
Total	395
Median Age (years)	40 (23–78)
BMI	22.85 (14.6–46.4)
Median NLR	1.85 (0.47–16.86)
Median PLR	8.89 (3.3–184.2)
Median LMR	4.34 (0.32–18.5)
HPV positivity	214 (94.3%)
p16 positivity	198 (79.8%)
after conization	
Smoking status	
Yes	63 (16%)
No	326 (83%)
N/A	6 (1%)
Alcohol consumption	
Yes	2 (0.5%)
No	386 (98%)
N/A	7 (2%)
Known Diabetes	
Yes	3 (1%)
No	392 (99%)
Known Hypertension	
Yes	37 (9%)
No	358 (91%)
Immunological disorders	
Yes	27 (7%)
No	368 (93%)

**Table 2:** Relationship between laboratory parameters and p16 positivity. The table shows the Mann–Whitney U test results for the association between p16 positivity in conization and hematological parameters, including neutrophil/lymphocyte ratio (NLR), platelet/lymphocyte ratio (PLR), and lymphocyte/monocyte ratio (LMR). * *p* < 0.05.

Laboratory Parameters	Mann–Whitney U	z	*p*
NLR	3687.5	−2.555	0.011 *
PLR	4308.0	−0.904	0.366
LMR	4064.5	−1.459	0.145

**Table 3:** Laboratory markers and correlation of HPV DNA status. Summary of laboratory markers (NLR, PLR, and LMR) in relation to HPV DNA status. The table displays the median, range, minimum, and maximum values for each marker across HPV DNA-negative and HPV DNA-positive groups. The results highlight higher median NLR and PLR values and lower median LMR values in the HPV DNA-positive group compared to the HPV DNA-negative group.

HPV DNA Status		NLR	PLR	LMR
Negative	Median	1.54	7.64	5.38
	N	12	12	11
	Range	1.39	5.88	2.94
	Min.	1.09	5.87	3.96
	Max.	2.48	11.75	6.90
Positive	Median	1.79	9.08	4.39
	N	213	212	212
	Range	16.39	155.71	17.90
	Min.	0.48	3.34	0.62
	Max.	16.86	159.05	18.52
Total	Median	1.76	8.93	4.46
	N	225	224	223
	Range	16.39	155.71	17.90
	Min.	0.48	3.34	0.62
	Max.	16.87	159.05	18.52

**Table 4:** Relationship between laboratory parameters and HPV positivity. The table shows the Mann–Whitney U test results for the association between HPV positivity and hematological parameters, including neutrophil/lymphocyte ratio (NLR), platelet/lymphocyte ratio (PLR), and lymphocyte/monocyte ratio (LMR).

Laboratory Parameters	Mann–Whitney U	z	*p*
NLR	988.0	−1.322	0.186
PLR	1013.0	−1.186	0.366
LMR	775.0	−1.874	0.061

**Table 5:** Association between laboratory markers and influencing factors. Statistical associations between laboratory markers (NLR, PLR, and LMR) and influencing factors such as age, smoking, alcohol consumption, hypertension, diabetes, and immunological disorders. * *p* < 0.05.

	NLR			PLR			LMR		
	Pearson Correlation		*p*	Pearson Correlation		*p*	Pearson Correlation		*p*
Age	0.074		0.145	0.038		0.458	−0.127		0.012 *
	U	z	*p*	U	z	*p*	U	z	*p*
Smoking	9421.5	−0.930	0.353	9663.0	−0.557	0.578	9504.0	−0.680	0.497
Alcohol consumption	0.0	−1.728	0.084	3.0	−1.700	0.089	1.0	−1.718	0.086
Hypertension	5056.5	−2.304	0.021 *	4901.0	−2.498	0.012 *	5374.5	−1.500	0.134
Diabetes	314.5	−1.376	0.169	547.5	−0.170	0.865	229.0	−1.801	0.072
Immunological disorders	4841.5	−0.104	0.917	4586.5	−0.511	0.610	4007.5	−1.501	0.133

## Data Availability

The data supporting the findings of this study can be obtained by contacting the corresponding author.

## Abbreviations

The following abbreviations are used in this manuscript:

AUC	Area Under the Curve
CIN	Cervical Intraepithelial Neoplasia
HPV	Human Papillomavirus
HR-HPV	High-Risk Human Papillomavirus
IHC	Immunohistochemistry
LEEP	Loop Electrosurgical Excision Procedure
LMR	Lymphocyte/Monocyte Ratio
NLR	Neutrophil/Lymphocyte Ratio
PLR	Platelet/Lymphocyte Ratio
ROC	Receiver Operating Characteristic
